# Predictors of vascular disease in myelodysplastic syndromes

**DOI:** 10.1002/jha2.101

**Published:** 2020-09-28

**Authors:** Mark G. Faber, David R. Lloyd, Abhay Singh, Jeffrey Baron, Amanda Przespolewski, Elizabeth A. Griffiths, Eunice S. Wang, Swapna Thota

**Affiliations:** ^1^ Roswell Park Comprehensive Cancer Center Buffalo New York USA; ^2^ Department of Medicine Jacobs School of Medicine and Biomedical Sciences State University of New York at Buffalo Buffalo New York USA

**Keywords:** atherosclerosis, hematopoiesis, myelodysplastic syndrome

## Abstract

The escalating link between somatic mutations commonly seen in myelodysplastic syndromes (MDS) and atherosclerotic vascular disease has increased the interest in management and associations of these conditions. We present a retrospective study examining clinical and molecular variables associated with vascular disease in patients with MDS. This study included a comprehensive evaluation of 236 patients with MDS. Our study has multiple findings. Mutations in ASXL1 correlated with increased risk of vascular disease for the entire cohort (*P* = .013). Though this has been replicated in other studies, there are no guidelines at this time for preventing vascular events in these patients. Our study also showed that lower ferritin levels may be linked to increased vascular events (*P* = .043), therefore the optimal use of supportive red blood cell transfusions in patients with MDS and the overall impact of inflammatory markers such as erythrocyte sedimentation rate and c‐reactive protein should be re‐addressed. Furthermore, our study showed that patients with Trisomy 8 in the low‐risk MDS cohort (based on IPSS‐R scores) were protected from vascular events (*P* = .036). Our findings of lower ferritin being linked with increased risk of vascular events as well as patients with Trisomy 8 being protected from vascular events may impact patient care. There do not appear to be any prior studies with these findings. In addition, given the connection between MDS and atherosclerotic vascular disease, we believe guideline‐based management of cardiac risk factors among MDS patients may improve overall outcomes. Further studies with larger patient cohorts are needed to further investigate these findings.

## 1 INTRODUCTION

Mutated hematopoietic cells lead to a bystander proinflammatory state resulting in increased risk of death from vascular diseases [1, 2, 3, 4]. Clonal hematopoiesis of indeterminate potential (CHIP) describes healthy individuals with a myeloid malignancy‐associated somatic mutation in blood without other diagnostic criteria for a hematologic malignancy [1]. The incidence of CHIP rises with age and is often associated with somatic mutations in *DNMT3A*, *TET2*, and ASXL1 [2, 3]. CHIP has unraveled a new mechanism for atherosclerotic vascular disease. Mutations in *ASXL1*, *TET2*, and *DNMT3A* have been linked to 1.9 times greater risk of coronary heart disease compared to non‐carriers of CHIP mutations [4].

Myelodysplastic Syndromes (MDS) and CHIP share common genomic events and are strongly associated with aging [5]. In fact, a subset of MDS cases is believed to arise from a preexisting CHIP state. A recent SEER study demonstrated an increased incidence of vascular events for MDS patients living 5 years beyond their MDS diagnosis [6]. These individuals had a likelihood of death attributed to vascular disease that approximated that of MDS itself [6]. Patients with low risk MDS were shown to have an elevated risk of death from cardiovascular disease [6]. As the link between vascular disease and clonal hematopoiesis strengthens, management of vascular disease in patients with MDS has become essential. It is key to establish which subset of MDS patients are at risk of death from vascular disease. Hence, we investigated both clinical and genetic factors predictive of vascular disease in patients with MDS.

## 2 METHODS

This retrospective analysis included 236 adult patients with a confirmed MDS diagnosis successively seen and treated at our academic medical center between 2010 and 2018. This study was approved by the Institutional Review Board of our institution. Each patient was ≥ 18 years of age with a diagnostic bone marrow biopsy reviewed at our center as well as detailed clinical information. Laboratory values were obtained as close to the time of diagnosis as possible. Cases were comprehensively evaluated for vascular events, defined as imaging or procedure‐verified coronary artery disease (CAD), cerebrovascular accident (CVA), or peripheral vascular disease (PVD). Additional details on blood counts, ferritin levels, and MDS classification and treatment details were obtained as closed to the time of diagnosis as possible. We calculated the Revised International Prognostic Scoring System (IPSS‐R) for patients based on data at or nearest in time to MDS diagnosis [7]. IPSS‐R has five risk groups; we used the five groups to define two main groups for this study. We defined low risk MDS as an IPSS‐R score <4.5 while defining high risk MDS as an IPSS‐R score of 5 and higher. Conventional karyotype information was present in 99% of the cohort. Next generation sequencing‐based multi‐gene sequencing results were evaluated in 37% (n = 87), with a majority using FoundationOne Heme assay (Foundation Medicine Inc, Cambridge, MA, USA), which detects mutations at minor allele frequency threshold of at least 5% [8]. An additional 66 patients (28%) had limited specific mutational data accounted for in our study.

## 3 RESULTS

**FIGURE 1:**
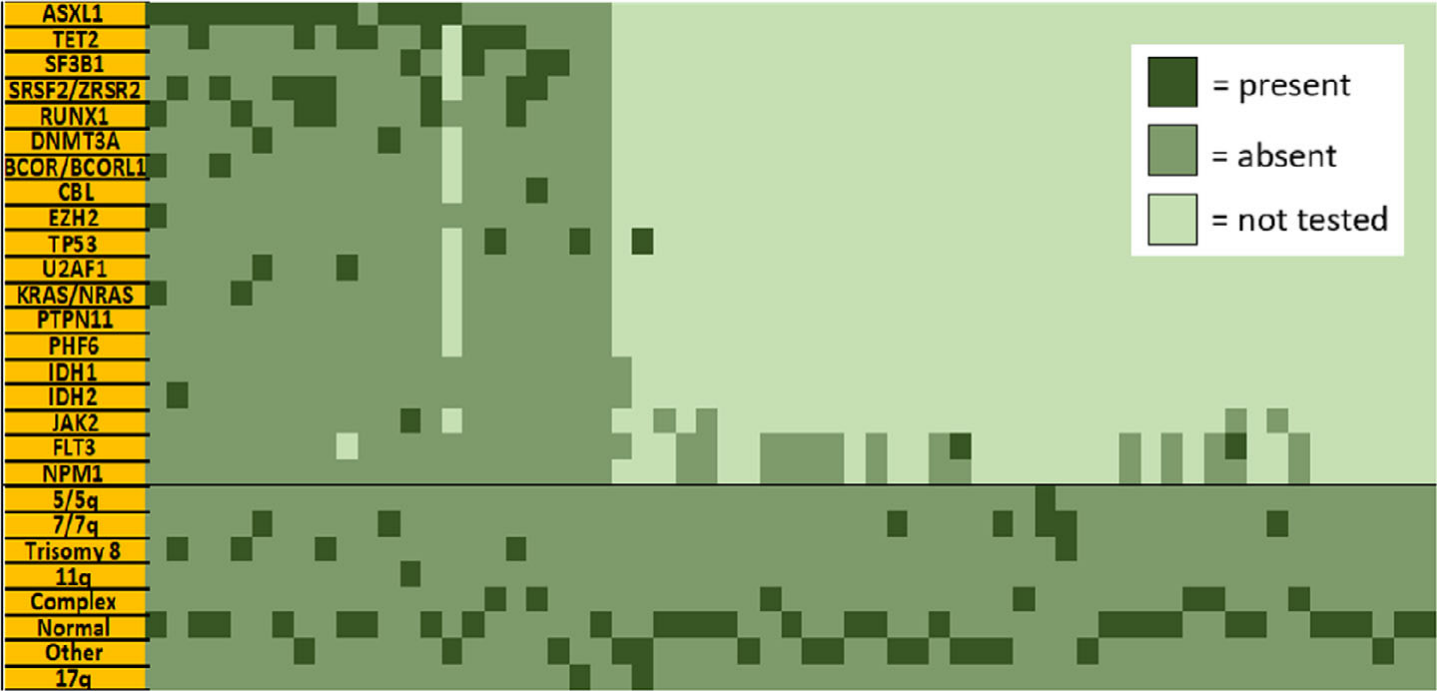
The figure represents a graphic depiction of mutations and cytogenetics for the entire cohort. The left shows 87 patients who underwent a full mutational analysis (mostly with FoundationOne). The right shows patients who had their cytogenetics analyzed with additional mutational analysis as noted

Median age at diagnosis was 69 (range: 20‐91) years; 61% were men in this cohort. Patient characteristics are shown in Table 1. By IPSS‐R, 8% had very low risk disease, 22.5% had low risk disease, 23.4% had intermediate risk disease, 24.8% had high risk disease, and 21.2% had very high risk disease. IPSS‐R scores were not calculated for 5.9% of the cohort due to unavailable data. The most observed karyotypic patterns were normal (46%), complex (17%), trisomy 8 (11%), del 7/7q (9%), and others (19%). Our analysis included eight of the nine CHIP mutations known to occur with greater than 1% occurrence – *SF3B1*, *SRSF2*, *JAK2*, *CBL*, *TP53*, *TET2*, *ASXL1*, and *DNMT3A* [9]. The most recurrent somatic mutations were *ASXL1* (40%), *SRSF2* (33%), *TET2* (32%), *SF3B1* (21%), *RAS* pathway genes (21%), *RUNX1* (21%), and *TP53* (15**%**) (Figure 1). We noted an overall incidence of vascular disease of 27%. Except for very low risk disease (5.1%), the incidence of vascular events by IPSS‐R categories was evenly distributed between low (23.7%), intermediate (22%), high (25.4%), and very high (23.7%) risk disease. Among the 63 MDS patients with one or more vascular events, 55 had documented CAD, seven had CVA, and 10 had PVD. Furthermore, among the 63 patients who suffered vascular events, 20% of their vascular events occurred post MDS diagnosis; of these, five patients had vascular events before and after the time of diagnosis.

**TABLE 1:** Baseline characteristics of the patients with and without a vascular events

		Vascular MDS (n = 63)	Non‐vascular MDS (n = 173)	*P*‐value
Mean Age		71.8	65.5	.001
Age Range		52 to 91	20 to 89	
Gender	Male	51 (81%)	94 (54.3%)	.001
**Dx**				
Low Risk MDS		30 (50.8%)	90 (55.2%)	.648
High Risk MDS		29 (49.2%)	73 (44.8%)	
**IPSS‐R**				
1 (very low risk)		3 (5.1%)	15 (9.3%)	.864
2 (low risk)		14 (23.7%)	36 (22.2%)	
3 (intermediate risk)		13 (22%)	38 (23.5%)	
4 (high risk)		15 (25.4%)	40 (24.7%)	
5 (very high risk)		14 (23.7%)	33 (20.4%)	
**Cytopenias**				
Hemoglobin (gm/dl)		9.87	9.68	.53
Hb Range		4.5 to 15.0	4.9 to 17.1	
Marrow Blast (%)		7.3	6.3	.2
Marrow Blast Range		0 to 20	0 to 22	
Ferritin ‐ Mean (ng/ml)		580.4	1006	.132
Ferritin ‐ Median (ng/ml)		283	464	
Ferritin – Square Root		19.8	26	.043
Elevated Ferritin (%)		24 (55.8%)	93 (75.6%)	.02
Low Ferritin (%)		19 (44.2%)	30 (24.4%)	
**Risk Factors**				
Smoking		32 (51.6%)	91 (53.2%)	.882
Non‐smoking		30 (48.4%)	80 (46.8%)	
DM		16 (25.4%)	27 (15.6%)	.09
Non‐DM		47 (74.6%)	146 (84.4%)	
HTN		40 (65.6%)	82 (47.4%)	.017
Non‐HTN		21 (34.4%)	91 (52.6%)	
HLD		38 (60.3%)	66 (38.2%)	.003
Non‐HLD		25 (39.7%)	107 (61.8%	
**CHIP Mutation Analysis**		**n = 22**	**n = 67**	
Mean CHIP Mutations		1.82	1.49	.176
CHIP Mutation Present		21 (95.5%)	56 (83.6%)	.280
TET2 Mutation Present		8 (36.4%)	20 (29.9%)	.603
ASXL1 Mutation Present		14 (63.6%)	22 (32.8%)	.013

**FIGURE 2:**
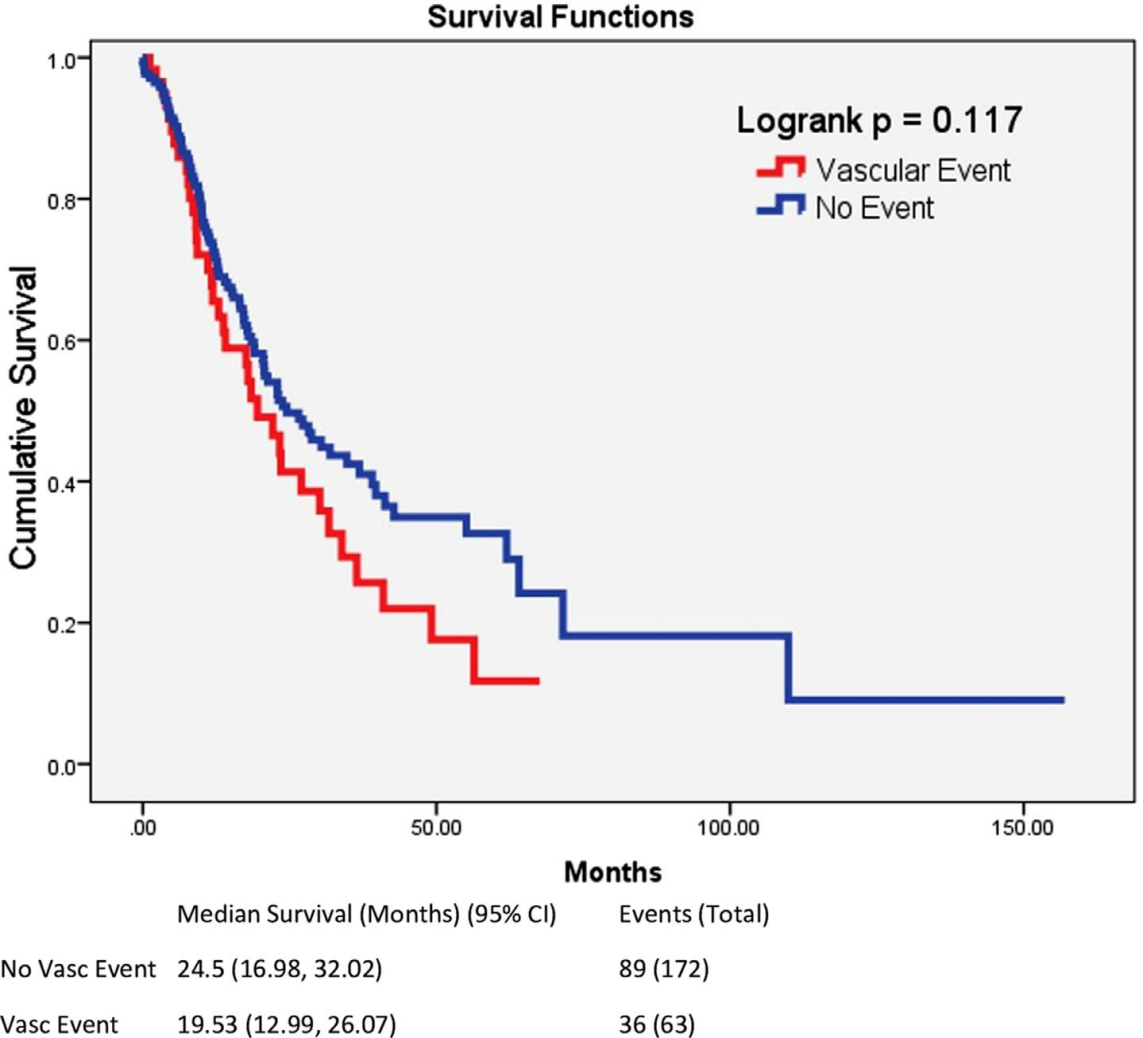
The figure shows cumulative survival of the patients with and without a history of at least 1 vascular event. The red line represents those patients with a prior vascular event and the blue line represents those without a prior vascular event

We noted several findings of interest. Expectedly, among the entire cohort, patients with vascular disease had a trend towards lower overall survival of 19.5 versus 24.5 months (*P* = .117) (Figure 2). In univariate analysis, women were significantly less likely to suffer vascular events than men (13% vs 35%, *P* < .001). The mean age of patients with vascular disease was also higher (72 vs 66 years, *P* = 0.001). Baseline blood counts and blast percentage were comparable. MDS patients without vascular disease had a significantly higher frequency of elevated ferritin levels, compared to those with vascular disease (76% vs 56%, *P* = .020) and based on the upper limit of normal of 200 ng/mL, consistent with American College of Physicians reported normal values [10]. The mean ferritin in the non‐vascular group was greater than in the vascular group (1006 vs 580) however large variability in ferritin values resulted in a non‐statistically significant difference (*P* = .132). Reducing variability by using the square root of the ferritin value has been proposed by prior authors [11] and resulted in a statistically significant difference (*P* = .043). Treatment with drugs typically associated with prothrombotic states, such as erythropoietin‐stimulating agents and lenalidomide, as well as other therapies such as hypomethylating agents were not associated with increased vascular events albeit limited sample size.

In the univariate analysis for the entire cohort, ferritin levels (OR: 2.54, *P* = .016), male gender (OR: 3.572, *P* < .01), and mutations in *ASXL1* gene (OR: 3.58, *P* = .013) were the variables predictive for vascular disease that are not traditional cardiac risk factors (Table 2A). We assessed low and high risk MDS populations for vascular disease risk factors, as separate cohorts. Among low risk MDS patients, male sex and hyperlipidemia were associated with vascular disease (Table 2B). Trisomy 8 was associated with a lower incidence of vascular disease in the low risk cohort (*P* = .036) as was elevated ferritin (*P* = .013). In the high‐risk disease group, traditional risk factors such as hyperlipidemia, hypertension and age were the most strongly predictive for vascular events (Table 2C). A multivariable analysis controlling for age, molecular events, and ferritin values only demonstrated a significantly increased risk of vascular events with advanced age (1.106,CI:1.015 to 1.206, *P* = .021) and *ASXL1* mutations (4.236,CI:1.083 to 16.579, *P* = .038) (Table 3).

**TABLE 2:** Associations of MDS variables with vascular events. (A) shows data for the entire cohort of MDS patients, (B) shows data for patients with MDS with low risk of progression, and (C) shows data for patients with MDS with high risk of progression

(A) All patients (n = 236)			
Variable	Comparison	Odds Ratio (95% CI)	*P*‐value
Age	Age vs 1 year younger	1.054 (1.023 to 1.086)	.001*
Sex	Male vs Female	3.572 (1.780 to 7.167)	<.001*
Ferritin	Under 200 vs Over 200	2.454 (1.184 to 5.088)	.016*
Hyperlipidemia	Present vs Absent	2.464 (1.365 to 4.448)	.003*
Diabetes	Present vs Absent	1.841 (0.914 to 3.708)	.088
HTN	Present vs Absent	2.114 (1.152 to 3.877)	.016*
Smoking History	Present vs Absent	0.938 (0.524 to 1.678)	.828
ASXL1 Mutation	Mutation vs Normal	3.580 (1.307 to 9.801)	.013*
TET2 Mutation	Mutation vs Normal	1.446 (0.519 to 4.028)	.480

(B) Low risk MDS (n = 120)			
Variable	Comparison	Odds Ratio (95% CI)	*P*‐value
Age	Age vs 1 year younger	1.043 (1.002 to 1.086)	.038*
Sex	Male vs Female	5.687 (1.835 to 17.628)	.003*
Ferritin	Under 200 vs Over 200	3.537 (1.341 to 9.329)	.011*
Hyperlipidemia	Present vs Absent	2.474 (1.054 to 5.806)	.037*
Diabetes	Present vs Absent	2.364 (0.859 to 6.503)	.096
HTN	Present vs Absent	1.932 (0.813 to 4.588)	.136
Smoking History	Present vs Absent	1.282 (0.560 to 2.938)	.557
ASXL1 Mutation	Mutation vs Normal	2.900 (0.741 to 11.347)	.126
TET2 Mutation	Mutation vs Normal	1.562 (0.389 to 6.269)	.529

(C) High risk MDS (n = 102)			
Variable	Comparison	Odds ratio (95% CI)	*P*‐value
Age	Age vs 1 year younger	1.065 (1.013 to 1.119)	.013*
Sex	Male vs Female	3.162 (1.152 to 8.681)	.025*
Ferritin	Under 200 vs Over 200	1.267 (0.335 to 4.784)	.727
Hyperlipidemia	Present vs Absent	2.788 (1.147 to 6.775)	.024*
Diabetes	Present vs Absent	1.341 (0.478 to 3.760)	.577
HTN	Present vs Absent	3.230 (1.229 to 8.490)	.017*
Smoking History	Present vs Absent	0.587 (0.243 to 1.415)	.235
ASXL1 Mutation	Mutation vs Normal	2.600 (0.518 to 13.041)	.245
TET2 Mutation	Mutation vs Normal	1.357 (0.202 to 9.126)	.753

**TABLE 3:** Removing sex as covariate. This is a multivariate analysis of MDS variables controlling for age, molecular events, and ferritin values with gender excluded

Variable	Comparison	Odds ratio (95% CI)	*P*‐value
Age	Age vs 1 year younger	1.106 (1.015 to 1.206)	.021*
Ferritin	Under 200 vs Over 200	0.408 (0.106 to 1.573)	.193
ASXL1 Mutation	Mutation vs Normal	4.236 (1.083 to 16.579)	.038*
Trisomy8 Mutation	Mutation vs Normal	0.720 (0.98 to 5.278)	.746

## 4 DISCUSSION

As the link between CHIP and atherosclerosis deepens, management of vascular disease is becoming a key clinical issue among patients with MDS. It is important to note that these unique characteristics were specific to MDS IPSS‐R classification categories. Established modifiable risk factors for vascular disease including hypertension and hyperlipidemia were key factors among high and very‐high risk IPSS‐R MDS patients, while in low risk MDS patients, hyperlipidemia was significantly linked to vascular events [12]. Here, we identify characteristics in MDS patients uniquely associated with increased vascular disease risk aside from traditional risk factors. We have comprehensively annotated cases for all known atherogenic risk factors including smoking as well as MDS disease features in comparison to the other published studies [7, 8]. In the low risk MDS cohort, ferritin is significantly decreased in those with vascular disease, likely representing the patient cohort with lower number of cumulative blood transfusions since diagnosis. Upon analysis of the entire cohort, the presence of *ASXL1* mutations was linked to increased odds of experiencing a vascular event. Treatment of MDS with erythropoietin injections or lenalidomide was not associated with an increased risk for vascular events, likely to due to limited sample size.

Two retrospective studies (N = 77 [13] and N = 566 [14]) were conducted at outside institutions to study the link between MDS and cardiovascular disease. In these studies, associations were established for *TP53* mutations with smoking (32% vs 15%, *P* = 0.10) [13] and cardiovascular disease (HR: 25.8, 95% CI: 1.96‐340.5, *P* = .013) [13] as well as *DNMT3A* mutations with a history of myocardial infarction (14% vs 6%, *P* = .03) [14]. Our study did not replicate these findings, as only *ASXL1* mutations were significantly linked to cardiovascular events.

Our results confirm that management of traditional cardiac risk factors among MDS patients such as hyperlipidemia and hypertension may improve their overall outcomes. However, heightened attention may need to be given to MDS patients with older age and somatic mutations in *ASXL1*. The optimal use of supportive red blood cell transfusions in patients with MDS may also be re‐addressed given our data that lower ferritin levels are linked to increased events. The impact of inflammatory markers such as ferritin as well as molecular events needs to be explored and validated in larger patient cohort.

## CONFLICT OF INTEREST

The authors declare no conflict of interest.
